# Deprivation in England, 1971–2020

**DOI:** 10.1007/s12061-022-09486-8

**Published:** 2022-11-16

**Authors:** C. D. Lloyd, P. D. Norman, D. McLennan

**Affiliations:** 1 Geography, School of Natural and Built Environment, Queen’s University Belfast, Belfast, UK; 2 School of Geography, University of Leeds, Leeds, UK; 3 deprivation.org, Hove, UK

**Keywords:** Deprivation, Inequalities, Census, Population change

## Abstract

Measures of small area deprivation have played a major role in targeting resources in the UK. The English Index of Multiple Deprivation (IMD) is the official measure of small area deprivation in England and it has been used to allocate billions of pounds of government money. The success of schemes to reduce deprivation can only be assessed by measuring changes in deprivation over time. In addition, the effect of such schemes is likely to be a partly a function of the deprivation history of an area. More generally, the trajectory of deprivation, and not just its current state, is important in understanding the likely impacts of deprivation on those who live in deprived areas. This paper combines the strengths of the IMD as a broad-ranging measure based on administrative data (here, using the 2004, 2007, 2010, 2015 and 2019 indices) and the Townsend score derived from Census data for a much longer time period (1971 to 2011). In addition, benefit claimant count data are used as a proxy for unemployment following the national Covid-19 lockdowns. The paper identifies some major trends in small area deprivation and unemployment over the period 1971 to 2020 and it highlights some key similarities and differences between the Townsend score and the IMD and makes links to changes in unemployment in 2020. Areas with very long term deprivation are identified and the strong association between job losses following Covid-19 lockdown and deprivation histories is demonstrated. The analyses are used to argue that deprivation trajectories should be considered if effective strategies for reducing spatial inequalities are to be developed.

## Introduction

The measurement of area deprivation has attracted considerable attention over the last 30 years and composite measures have played a major role in identifying areas in need and in allocating financial and other resources; in the UK, the English Index of Multiple Deprivation (IMD) (see Noble et al., 2006) and its equivalents in Wales, Scotland and Northern Ireland, are used to allocate billions of pounds of public money annually. Numerous measures of deprivation have been proposed and in Britain these have traditionally tended to make use of data from the Census (see Senior, 2002 for a review). More recently, these national indices of multiple deprivation across England, Wales, Scotland, and Northern Ireland, have become the key means for profiling deprivation in small areas of the UK. It has been estimated that as much as 1% of all government spending in England was allocated using the IMD.^1^ The IMDs (note that different terminology is used at different time points and in different nations, but IMD is used here throughout) for each of the constituent nations of the UK cannot be directly compared although Abel et al. (2016) present a methodology which allows for adjustment of the income and employment domains of the IMD so that cross-UK comparisons can be made. Changing definitions mean that direct comparisons across time within countries are also problematic. Here it is argued that focusing on the state of deprivation at an individual time point is not sufficient and that an understanding of deprivation *trajectories* is essential both in understanding the challenges faced within local communities and in developing approaches to tackle spatial inequalities. Given this focus is it necessary to develop approaches to measuring and analysing deprivation change with the objective of better understanding how far neighbourhoods have long histories of deprivation, or only more recent socio-economic downturns. The exacerbation of inequalities demonstrated following the UK national Covid-19 lockdowns (see Adams-Prassl et al., 2020) has prompted an urgent need to better understand why some communities are ‘left behind’ and to develop more targeted approaches to helping those people and communities most affected by a growing cost of living crisis in the UK. The ‘levelling up’ agenda (HM Government, 2022) is a major driver for UK Government and it is here argued that a knowledge of deprivation trajectories is crucial in achieving any meaningful reduction of spatial inequalities.

Previous work on change in deprivation in Britain over small areas has utilised standard Census geographies with counts reallocated from source zones to a common target geography (e.g., wards). Norman et al (2003) and Norman (2010) explore change in deprivation using data for wards for 1991 and 2001. This work is extended by Norman (2016) and Norman and Darlington-Pollock (2017) who chart changes in deprivation in Britain between 1971 and 2011. Lloyd et al. (2017) characterised change in deprivation in Britain between 1971 and 2011 over 1 km grid cells developed as part of the PopChange project. Hincks (2015) utilises the 2004 IMD as a benchmark along with data (for 2001 to 2007) on the total population, benefits claimants and median house prices, to profile change in deprived areas of Greater Manchester. Profiling deprivation *change* is arguably as important as capturing deprivation at some recent time point. The 1971 to 2001 deprivation scores developed by Norman and colleagues have been utilised in analyses of changing cancer registrations and survival from cancer (for example, McNally et al., 2012). Other studies which explore deprivation change include those of Noble et al. (2001), who assessed geographic patterns of income deprivation, Evans et al. (2002), with a focus on temporal trends in benefit claimant rates, Barnes et al. (2011), who were concerned with worklessness and deprivation, and McLennan et al. (2012), focusing on economic deprivation in England between 1999 and 2009. In these studies, individual-level linked administrative data were used to explore the individual-level dynamics that underpinned the observed change (or lack of change) at small area level. It is worth noting that an area which appears to have 'no change' in deprivation might in fact have a high degree of change at the individual person level, with people moving into and out of poverty and people moving into and out of the area. Understanding the individual level dynamics in an area is extremely important in tailoring polices to tackle persistent deprivation (see Robson et al., 2008). Barnes et al. (2011) built on the Robson typology using individual level benefits administrative data to track people into/out of work and between geographical areas. Mobility into and out of areas is not considered in this study, but linking (for example), Census data on migration flows would comprise an informative additional element of future work.

The present study is focused on England and provides detailed comparisons of what can be captured about change over time using the Townsend Index (Townsend et al., 1988), the IMD, and claimant count data. Benefit claimant count^2^ data provide estimates of unemployment in LSOAs. The data used are experimental claimant counts (counts of Job Seeker’s Allowance and Universal Credit claimants^3^) and mid-year population estimates as denominators (provided by ONS) to derive estimates of unemployment levels. The IMD is used as it is the official measure of deprivation in England, while the Townsend index is used to provide context over a 40 year time period and benefit claimant count is used to provide a recent perspective.

The Townsend index incorporates information on unemployment, household overcrowding, housing tenure, and car or van ownership. The index is presented as a composite of the four indicators, created by converting the variables to *z* scores and summing the products (see below for more details). The value of the index lies in the ability to compute it using UK Census data for multiple time periods. It is possible, therefore, to track changes in deprivation for small areas over a long period of time (here, 1971 to 2011). The IMD, in contrast, comprises seven distinct domains of deprivation (defined below) which can be combined to provide a single measure. The Townsend score is derived from variables which can be used to chart absolute change (e.g., change in unemployment rates). The IMD comprises variables which are expressed as ranks and changing definitions and variables used in construction of the various releases of the index mean that only relative changes in the domains can be assessed.

This paper builds on previous work using purpose-specific methods for consistent Lower Super Output Area (LSOA) generation (linking the Townsend score and additional Census variables for 1971, 1981, 1991, 2001, 2011 and IMD for 2004, 2007, 2010, 2015, and 2019 and benefit claimant count data for 2020 to a common geography (the 2011 definition of the LSOAs). The paper seeks to answer the question of how far high levels of deprivation persist in small areas of England. Specifically, it aims to assess how far the two deprivation measures (IMD and Townsend) show similar features in terms of the most deprived areas. Profiling deprivation using different sets of measures in this way over a long time period could provide invaluable input into schemes intended to target deprivation. In short, strategies used in areas with persistent deprivation might be different to those with, for example, a recent downturn in employment. In addition, the success of previous schemes can be examined by measuring change in deprivation – a crucial element missing from most analyses of deprivation. Core questions the paper seeks to address include:

- How persistent is deprivation using the Townsend score?
- How persistent is deprivation using the IMD?
- How far do recent (post Covid 19 national lockdown) changes in claimant count rates correspond to high deprivation levels?
- Do the measures identify common areas with high (and persistent) deprivation?
- How far is persistence of high deprivation using the IMD predicted by high deprivation using the Townsend score and IMD in previous years?

The resource utilised here enhances previous work (e.g. Norman, 2016) by broadening the array of Census variables to include, for example, tenure breakdowns, number of rooms, and number of dwellings  (see Lloyd and Gleeson, 2022), along with the IMD for four time points. All of the data (Census counts and IMDs) are available through the PopChange data resource.^4^ The data used in the study are detailed first, followed by an outline of the Townsend score and the IMD. Next, an analysis of change in deprivation using the Townsend score is detailed, and then an equivalent based on the IMD. Following this, the two indices are combined in an assessment of long-term deprivation trends. Finally, changes in claimant count rates since the Uk national Covid-19 lockdowns are related to deprivation levels prior to lockdown.

## Data

**Fig. 1:**
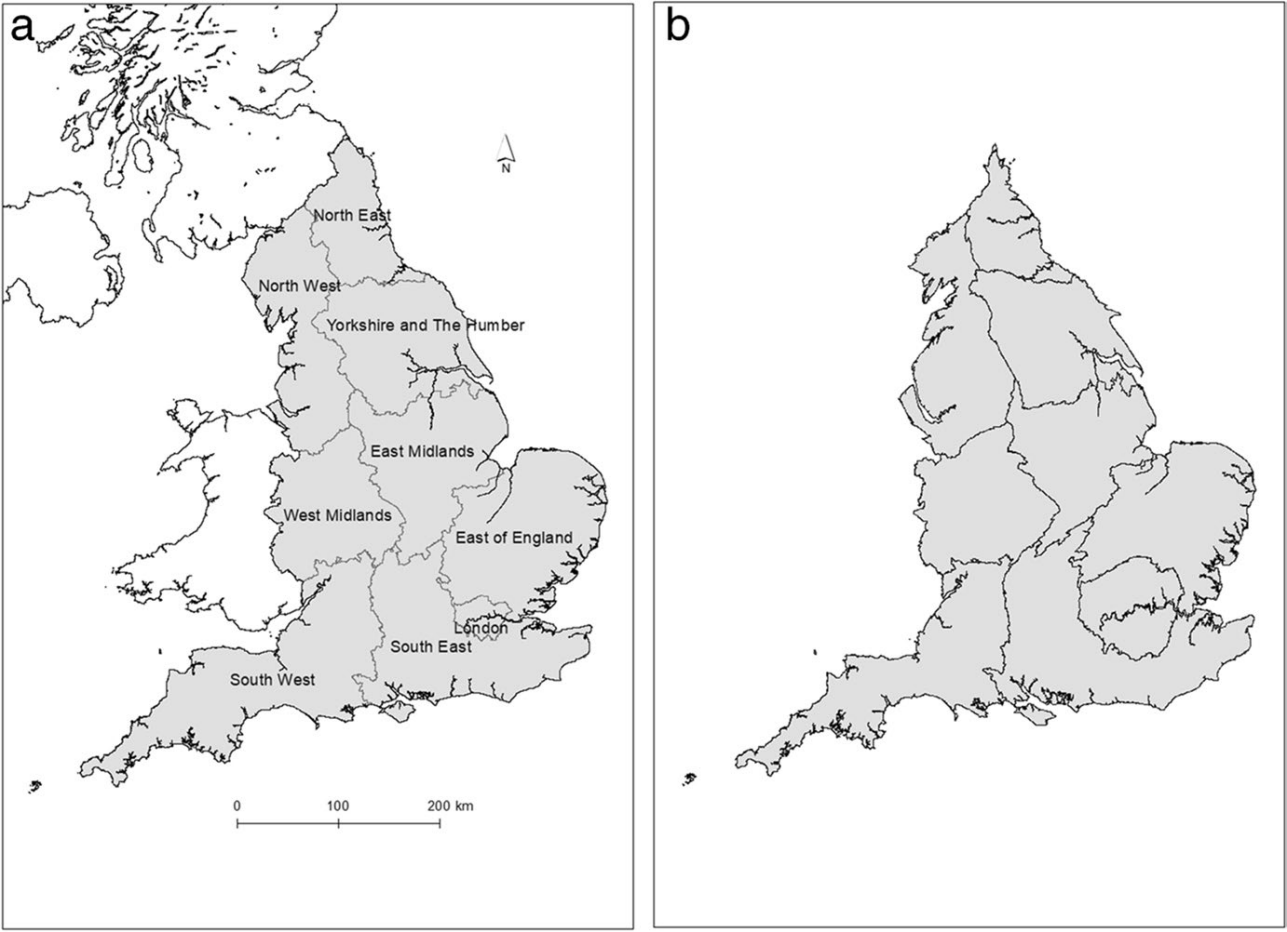
Regions of England in (**a**) normal map space, (**b**) as a cartogram

The analyses are based on Census data, the IMD, and claimant count rates for 2011 LSOAs in England (*n* = 32,844). LSOAs were designed so that their populations would be reasonably constant. This means that LSOAs in urban areas tend to be much smaller in urban areas than in rural areas. As a result, with maps of (for example) deprivation scores produced using LSOAs it can be difficult to discern patterns in urban areas. In this paper, rather than using standard map space, cartograms (Dorling, 1996) are used to distort physical space such that urban LSOAs appear relatively larger and rural LSOAs appear relatively smaller. The LSOAs are scaled by the square root of their original areas, in line with Harris (2016) and Harris et al. (2017). This achieves a balance between obscuring features in urban areas (the case using the data in their original map space) and excessive distortion resulting in very difficult to interpret maps. In practice, the LSOAs were generalised before construction of the cartograms as the computational load with the original data made the derivation of cartograms problematic. The new maps provide a much clearer indication of spatial and temporal trends in deprivation in urban areas. The regions of England are superimposed on the maps; this was achieved by dissolving the boundaries of distorted LSOAs allocated to a specific region. Figure 1a shows the regions in ordinary map space while the accompanying map (Fig. 1b) shows the regions in distorted space (that is, as a cartogram).

The Census data Tables used as inputs to the Townsend deprivation index are detailed in Table 1 see “Discussion and conclusions”.

**Table 1:** Townsend deprivation index Census data tables

Variable	1971	1981	1991	2001	2011
Employment	SAS05	SAS09	SAS08	KS009a	KS601
Tenure	SAS18	SAS10	SAS20	KS018	KS402
Overcrowding	SAS18	SAS10	SAS23	CS052	QS409
Car or van access	SAS18	SAS10	SAS20	KS017	KS404

### Reallocating Counts to 2011 LSOAs

The analysis is based on deprivation scores for 2011 LSOAs, as this is the geography used to disseminate counts from the 2011 Census and it is also used to report the most recent IMD. The Census data for 1971, 1981 and 1991 (for enumeration districts (EDs^5^)) were linked to 2011 LSOAs using postcodes to determine the weights for source zone – 2011 LSOA areas of intersection. Postcodes provide a proxy for population density within these overlapping areas, thus allowing more accurate assignment of population counts than would be possible using standard areal weighting. The 2001 LSOA counts were assigned to 2011 LSOA using the lookup table provided by the Office for National Statistics. The lookup was considered suitable since only some 2.5% of the 2001 LSOAs were changed as a result of the 2011 Census and so a more complex postcode-based lookup was unnecessary.

In the case of 1971, 1981 and 1991 Census data, the following procedure was followed:

1. Source (e.g., ED) and target (2011 LSOA) zones were overlaid.
2. Postcodes for the closest available time point were overlaid with the outcome of stage 1, giving account of postcodes in areas of intersection – this serves as a weight used as detailed below.
3. All areas of intersection with a postcode count of zero are given a weight of 0.1 to ensure that all source zones contribute to the reallocated counts and that all LSOAs are populated at the end of the process.
4. The areas in metres squared of the areas of intersection are multiplied by the weights (0.1 base value or postcode count)
5. The sum of weights × areas are aggregated by source zone
6. The output from 5 is joined to the areas of intersection using the source zone code
7. Estimates (of persons or households) within each area of intersection are obtained with weights × areas / sum(weights × areas) × Persons or Households in source zones.

ED boundaries for 1971 are not available and so instead 1971 ED centroids were spatially linked to 1981 EDs, as they are the closest available zones in date. EDs for 1981 which did not contain a 1971 ED centroid were merged with their closest 1981 ED which did contain a 1971 ED centroid, creating a set of merged 1981 EDs containing the total counts of all intersecting 1971 EDs (judged by centroid location). A similar process was followed by Lloyd et al. (2017; more on processing of Census and postcode data is also provided in that paper). For linking 2001 LSOAs to 2011 LSOAs the ONS best-fit lookup table^6^ was used. Only some 2.5% of 2001 LSOAs in England and Wales were changed for the 2011 Census (Tait, 2012) and so the strategies employed were considered sufficient.

### Deprivation Scores Derived from Census Data

The Townsend deprivation score (Townsend et al., 1988, and see Senior, 2002) is constructed using four sets of percentages:

- Unemployed persons (% of employed plus unemployed)^7^
- Non owner-occupied households (% total households)
- Households without access to a car or van (% total households)
- Households with more than one person per room (% total households)

In this analysis, the percentages of unemployed persons and households with more than one person per room (overcrowding) were logged (after addition of 1; this allows for the tendency for skewed distributions of these percentages). After this, the four variables (two percentages and two logged percentages) were converted to *z* scores ((percentage-mean) / standard deviation) and these were summed to derive deprivation scores. Positive values of the index indicate areas with higher levels of deprivation while negative values indicate lower levels of deprivation. It is worth noting that composite deprivation scores such as the Townsend score can be computed using z-scores based on (1) per-year means (e.g., the mean unemployment % for 2011) or (2) the mean across all years (e.g., the mean unemployment % for all Census years from 1971 to 2011 inclusive). In this case, the former approach is used.

### Index of Multiple Deprivation

The principles behind the IMD and its construction are detailed by Noble et al. (2006). Four versions of the IMD are used in the study: those for 2004 (Noble et al., 2004), 2007 (Noble et al., 2008), 2010 (McLennan et al., 2011), 2015 (Smith et al., 2015), and 2019 (McLennan et al., 2019; Noble et al., 2019). The IMD comprises seven domains, each based on a set of indicators, and the weights applied in creating the overall IMD are as follows:

- Income Deprivation Domain 22.5%
- Employment Deprivation Domain 22.5%
- Health Deprivation and Disability Domain 13.5%
- Education, Skills and Training Deprivation Domain 13.5%
- Barriers to Housing and Services Domain 9.3%
- Crime Domain 9.3%
- Living Environment Deprivation Domain 9.3%

The weighting approach adopted for the IMD is therefore substantively different to the weighting approach in the Townsend Index. For the IMD, the weights were derived through theoretical considerations which were subsequently validated through a separate piece of research (Dibben et al, 2007), whereas in the Townsend Index all four indicators are effectively assigned equal weights.

The data included in each of the IMD 2019 domains are detailed by Noble et al. (2019) and McLennan et al. (2019). While the same domains and weights were used in all versions of the IMD since 2004, the specific methodologies used in their construction changed and thus they cannot be compared directly. Instead, comparison of ranks is the most appropriate approach. Smith et al. (2015) outline changes in the inputs to the individual domains between the 2010 and 2015 IMDs. It is important to note that the various versions of the IMD are based on input data for various time periods; in the case of IMD2015 “As far as is possible, each indicator is based on data from the most recent time point available; in practice most indicators in the Indices of Deprivation 2015 relate to the tax year 2012/13”. The IMD scores are relative and thus a reduction in IMD ranks in a specific area could indicate that deprivation has declined in that area relative to other areas or it has increased elsewhere. In the former case, absolute deprivation could have decreased at all locations, but to a greater degree in the selected area. Smith et al. (2015) note changes to variables used in domains between 2010 and 2015, and there were minor changes in the 2019 indicators reflecting changes in available data (Noble et al., 2019).

### Claimant Count Rates

Experimental claimant count (Job Seeker's Allowance and some Universal Credit claimants; see ONS, 2015) data provide a proxy for unemployment in LSOAs. These data are used to give a current perspective on unemployment in the context of the Covid-19 pandemic. Mid-year population estimates of working age (16–64) people (for 2019) are used to compute the rates. Claimant count will likely under-estimate unemployment as not all people out of work are able to claim benefits. However, eligibility rules are relatively consistent geographically, thus claimant count is a useful guide to relative unemployment levels (ONS, 2017) (Tables 1, 3, 4, and 5).

### Analysis of Change by Area Type

One approach to assessing broad changes in deprivation is to use an area classification; this allows consideration of, for example, how far deprivation has become more or less urban-focused. The 2011 ONS Output Area Classification (OAC) for Local Authorities (LAs) (ONS, 2014) is used here to summarise deprivation values. The OAC comprises three tiers: Supergroups, Groups and Subgroups and it is derived using a set of 59 demographic and socioeconomic variables. Here, the top tier classification (eight Supergroups at UK level, of which seven are found in England) is used. The LA (linked to LSOA) classification provides a broad characterisation of areas which helps to understand the distribution of deprivation across England. The percentage of LSOAs in each OAC supergroup is given in Table 2. Examples of areas included in some of the classifications are given in the text to aid understanding of the findings.

**Table 2:** Count and percentage of LSOAs in each OAC supergroup

OAC Supergroup	*n* LSOAs	% LSOAs
Business and Education Centres	4631	14.10
Coast and Heritage	1982	6.03
English and Welsh Countryside	6590	20.06
London Cosmopolitan	2825	8.60
Mining Heritage and Manufacturing	7137	21.73
Prosperous England	4894	14.90
Suburban Traits	4785	14.57
Total	32,844	100.00

## Townsend Score

Table 3 shows the percentage of people or households in each group for each Census year. The most notable trend is an increase in unemployment between 1971 and 1981 and a decline between 1991 and 2001. The reduction in the percentage of non-owner-occupied households from 1971 to 2001 is partly a function of purchase of council houses through the ‘right to buy’ scheme.^8^ The increase in non-owner-occupation between 2001 and 2011 in part may relate to ‘buy to let’. The no car and overcrowding variables steadily decrease over time. It is worth noting that the definition of 'unemployed' (or whether an individual considers themselves to be unemployed) is likely to be associated with the policies at a given time point. For example, as people progressively moved off Job Seeker’s Allowance and onto Incapacity Benefit (IB) / Severe Disablement Allowance (SDA)^9^ in the late 1990s, there was an apparent fall in the level of ‘unemployment’, but an accompanying rise in people unable to work due to sickness or disability. As such, the changes in unemployment level in an area might be partly dependent on the ‘type’ of worklessness in the area. As another example, when the changes to Income Support eligibility changed by reducing the age range of children for whom lone parents could claim eligibility, this will have had the effect of moving these lone parents off Income Support and onto (most likely) Job Seeker’s Allowance. These individuals would then have moved from ‘inactive’ in the labour market to ‘unemployed’. Clasen et al. (2006) provide a comparative assessment of transitions between labour activity and inactivity in the UK and Germany.

**Table 3:** Percentages in each group for England

	Unemployed*	Non owner occupied	No car or van	Overcrowded
1971	3.84	49.96	48.22	6.05
1981	10.04	42.40	38.58	3.39
1991	9.12	32.70	32.42	2.11
2001	5.21	31.28	26.84	1.89
2011	6.59	35.87	25.80	2.10

^*^For 1971: seeking work/(working + seeking work); for 1981–2011 inclusive: unemployed/ (employees + self-employed + unemployed)

**Fig. 2:**
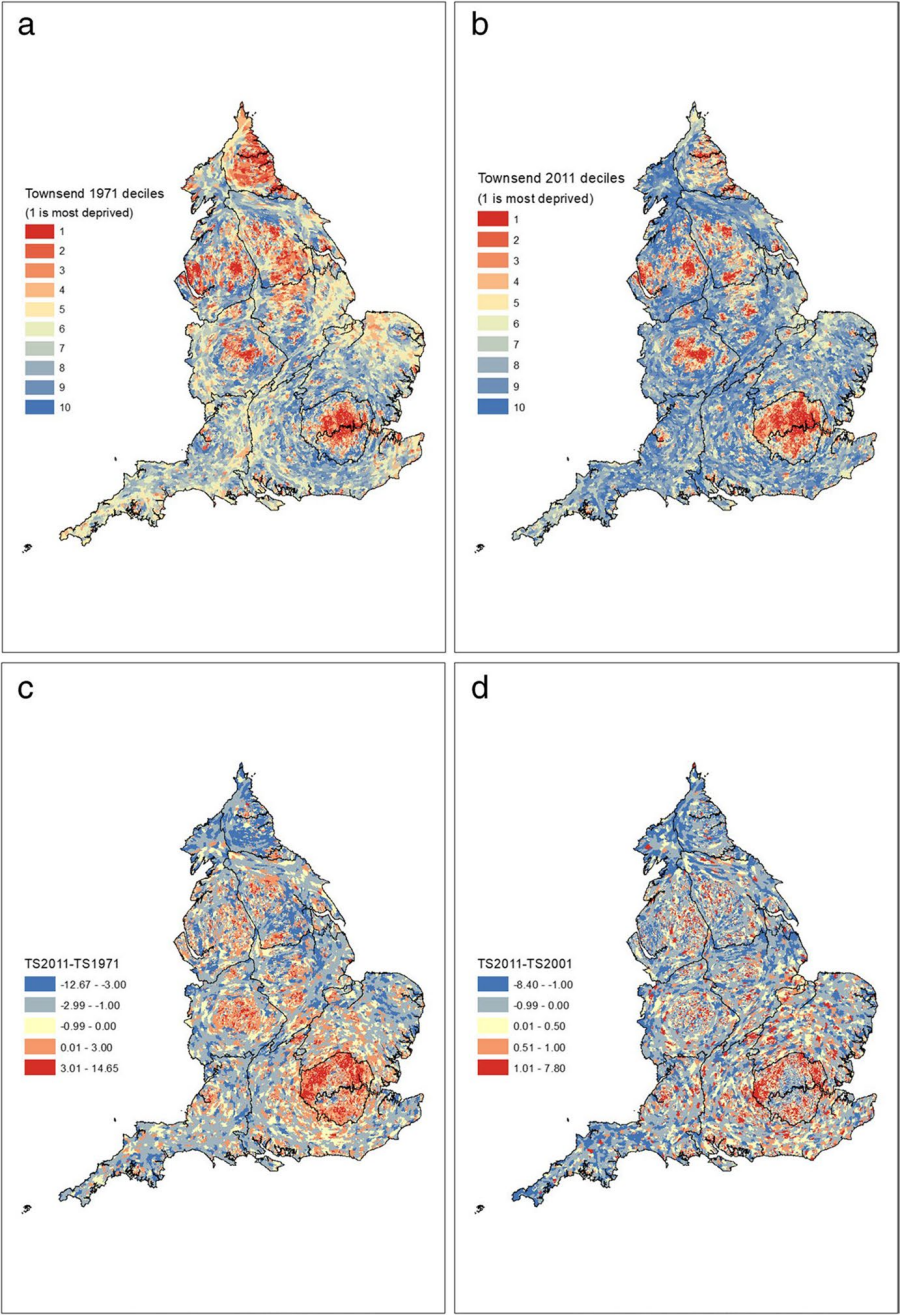
Townsend score in (**a**), 1971, (**b**) 2011, (**c**) 1971–2011, (**d**) 2001–2011

Figure 2 shows the Townsend score in (a), 1971, (b) 2011, (c) 1971–2011 (computed as 2011 score minus 1971 score, so that negatives indicate decreases and positives indicate increases in deprivation), (d) 2001–2011. Given that the index values for each year are derived using per-year means, the difference maps compare deprivation relative to one Census year (e.g., 1971) with deprivation relative to another Census year (e.g., 2011). The main geographical patterns in 1971 (Fig. 2a) and 2011 (Fig. 2b) are similar, with urban areas containing LSOAs in the most deprived deciles. An increased concentration of deprivation in urban areas between 1971 and 2011 is indicated by Fig. c, while Fig. 2d (2001–2011) suggests that while there were increases in relative deprivation in a ring around London, there is little evidence for strong geographical trends in changes elsewhere.

The trends inferred from the maps are supported by linking deprivation scores to Outputs Area Classifications (OACs) for 2011 – this shows that, in 1971, nearly 30% of the most deprived 10% of LSOAs were found in ‘London Cosmopolitan’ areas; in 2011 this figure had increased to almost 41%. Cross-tabulation of the deprivation deciles for 1971 and 2011 suggests that very few LSOAs moved from the highest to the lowest decile (and vice versa), but that there was a considerable amount of movement between other deciles, particularly in the middle ranges. The upper and lower deciles are bounded, so we would not expect as much movement in these as in the rest of the decile distribution. In addition, both the Townsend Index and the IMD measure *deprivation*, and not affluence. We know that many deprivation indicators are right-skewed so that the deprivation ranks will be more reliable at the upper end of the deprivation distribution (as small changes at the bottom end can, in theory, lead to large changes in rank within the less deprived end of the distribution).

**Fig. 3:**
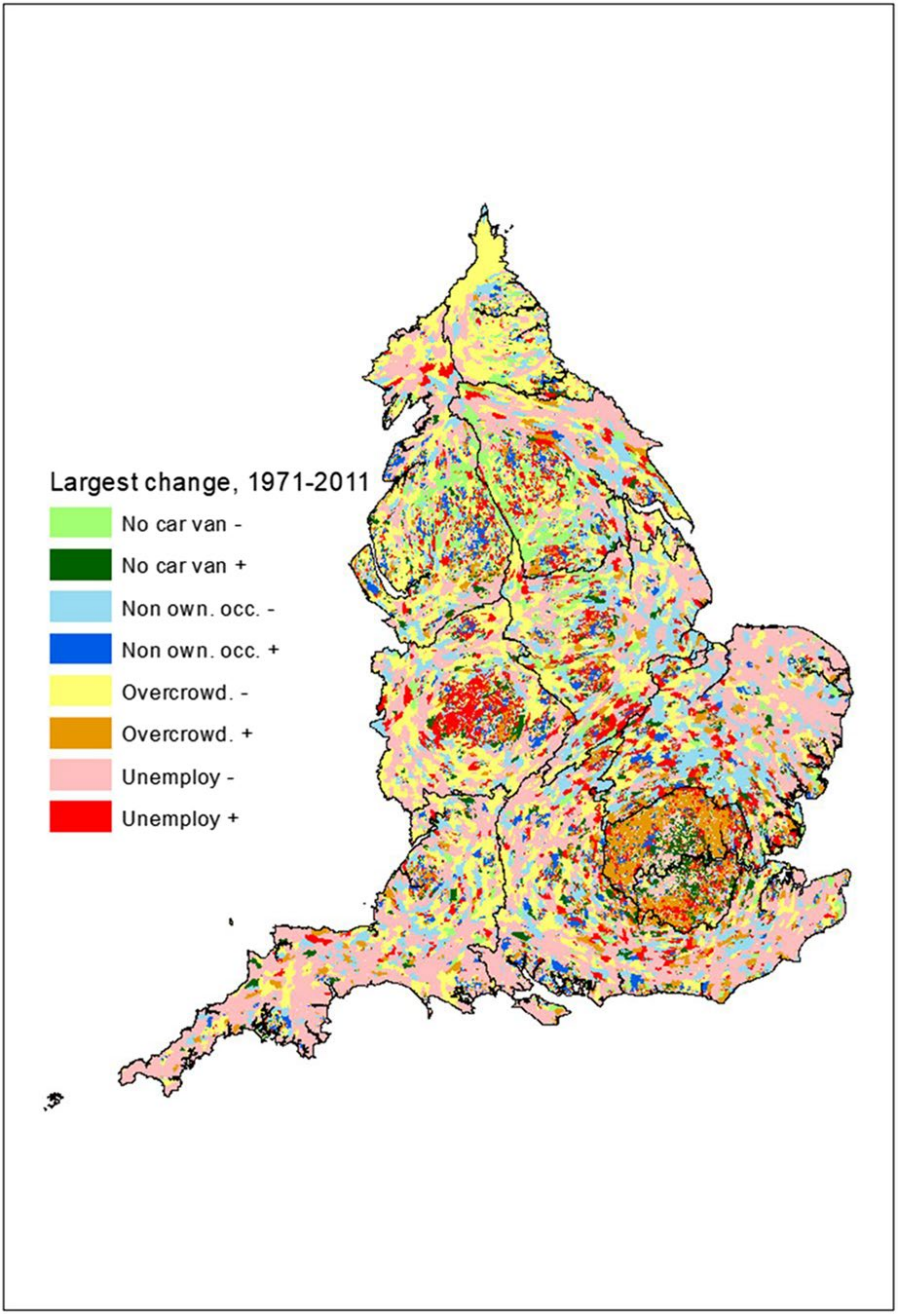
Largest change in z scores, 1971–2011

Changes in the Townsend score are a function of change in the four input variables – in this case, the weights are equal and so a direct comparison of the four scaled variables is meaningful. Contributions to changes in the Townsend score can be examined by identifying which of the four sets of *z* scores changed most between two time points. Figure 3 indicates which *z* scores changed most between 1971 and 2011 with each variable categorised as negative or positive; as an example ‘No car van –’ indicates that the largest change is a proportional decrease in No car or van access. The relative increase in overcrowding in the outskirts of London is a notable feature. The largest increases in overcrowding over this period are in neighbourhoods which saw increases in the proportions of people from Black, Asian and Minority Ethnic (BAME) groups. BAME groups are more likely to live in the private rented and social rented sectors, and these are the sectors associated with the increase in overcrowding (MHCLG, 2018; Lloyd & Gleeson, 2022; see also Stillwell, 2010). There is some evidence for a spread of deprivation outside of urban cores – as is the case for London, notably over the period 2001 to 2011. This ‘suburbanisation’ of deprivation is consistent with findings for British cities over the period 2004–16 (Bailey & Minton, 2018).

## Index of Multiple Deprivation

**Fig. 4:**
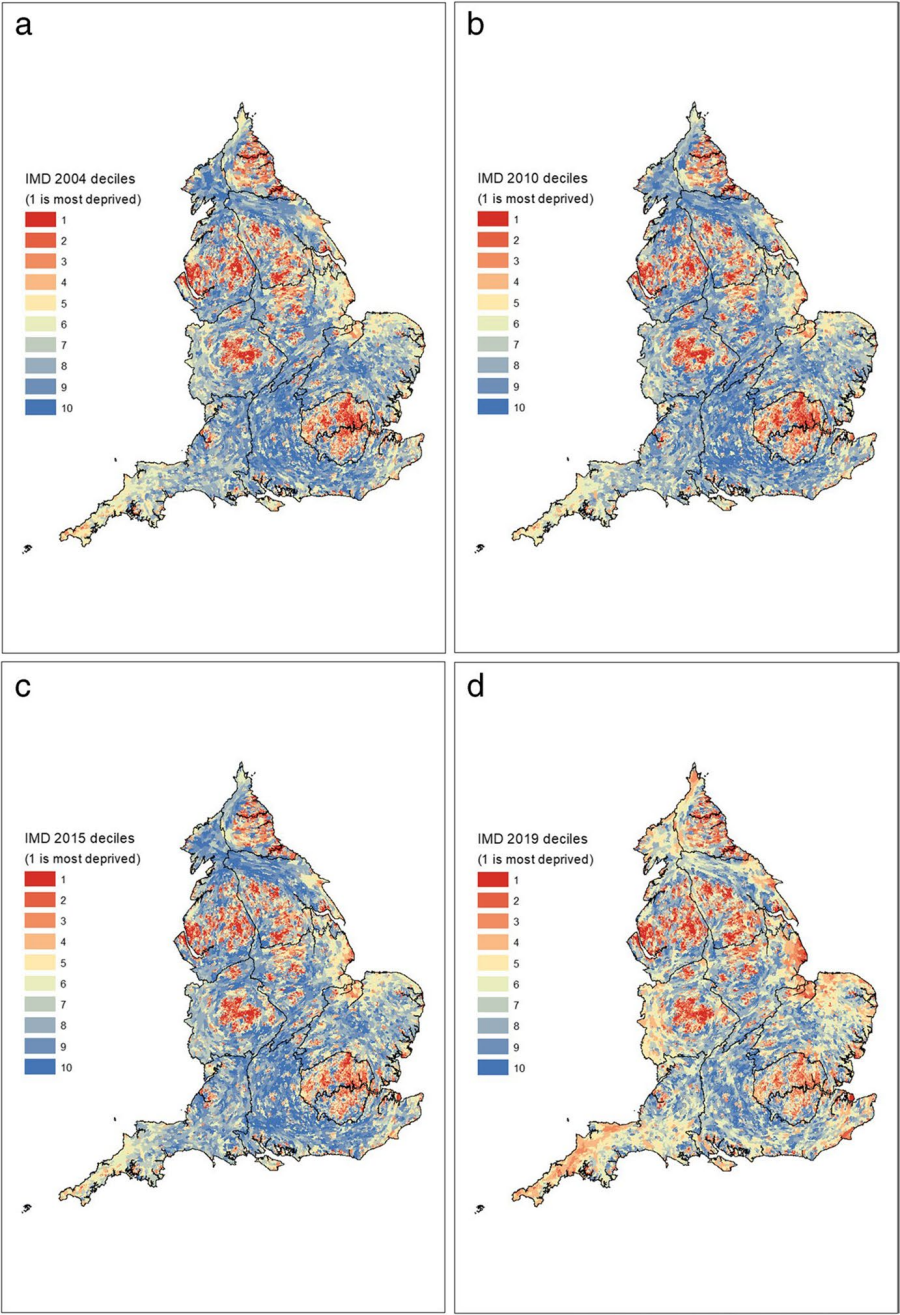
IMD in (**a**), 2004, (**b**), 2010, (**c**), 2015, (**d**), 2019

Very few LSOAs moved more than two deprivation deciles between 2004 and 2015, although there is greater movement between deciles in the middle range. This is expected given that the upper and lower ends of the distribution are bounded and scope for change tends to be smaller. Mapped IMD exhibits similar geographical trends to the Townsend score, with higher levels of deprivation in urban areas than elsewhere. Figure 4 shows the IMD for 2004, 2010, 2015, and 2019. Examination of mapped IMD and the share of LSOAs in the most deprived deciles suggests that deprivation was more consistent over the period 2004 to 2010 than the period 2010 to 2015, but the period from 2015–2019 was more consistent still. For example, some 82% of LSOAs in the top deprivation decile (the most deprived 10%) in 2004 remained in the top decile in 2010. Of those LSOAs in the top deprivation decile in 2010, 74% were still in the top decile in 2015. Of the LSOAS in the top decile in 2015, nearly 88% were also in the top decile in 2019. The 2010 IMD was based mainly on 2008 data – before the effects of the 2008 financial crash were felt – and so the fact that the period of greatest change was 2010 to 2015 likely reflects the impacts of the 2008 financial crash.

Over the period 2004 to 2019 there was a decrease in (relative) deprivation in central London and parts of other urban areas. Increases are found in many rural areas including parts of Cornwall and Devon, parts of East Anglia, southern parts of the Welsh border and the rural north west. The changes in ranks suggest a (relative) decrease in deprivation in much of London between 2010 and 2015, and in many cases this corresponds to a move of more than one deprivation decile (e.g., from decile 1 in 2010 to decile 3 in 2015). This is likely due to the lag effects of the 2008 financial crash, from which London bounced back more quickly than the rest of England, thereby representing a relative improvement in London compared to the rest of the country.

## Comparing Townsend Scores with the IMD

The TS and IMD exhibit the same broad patterns, with deprivation focused in urban areas. There is considerable consistency between the measures and across time. For example, some 42% of LSOAs in the top (i.e., the most deprived) TS71 decile were also in the top IMD19 decile. Some 79% of LSOAs in the top TS71 decile were in the top three deciles (the top 30%) by IMD19. There are, however, considerable differences between area types (represented by the OAC^10^) in the distribution of LSOAs in the top deprivation deciles. Table 4 shows the percentages of LSOAs in the top deprivation decile by OAC. The most obvious difference between the TS and the IMD is for the ‘London Cosmopolitan’ area type whereby TS71 and TS11 have much larger percentages (29.8% and 40.7% respectively) compared to the IMD04 (19.9%) and IMD19 (2.56%). The increase in overcrowding in outer London is a key factor in the increase in the TS percentage between 1971 and 2011. That the IMD focuses on different characteristics and that overcrowding has a small weighting is highlighted by the large decrease in the percentage of LSOAs in the top IMD deciles in London between 2004 and 2019.

**Table 4:** Percentages of LSOAs in top deprivation decile by OAC

Top 10%	Business and education centres	Coast and heritage	English and welsh countryside	London cosmopolitan	Mining heritage and manufacturing	Prosperous england	Suburban traits
TS71	27.28	2.34	4.32	29.80	28.01	0.94	7.31
TS11	28.19	2.25	1.04	40.73	13.33	0.37	14.09
IMD04	32.33	3.44	3.62	19.85	28.98	0.58	11.20
IMD19	35.37	6.03	6.54	2.56	38.78	0.70	9.98

Table 5 shows the top 10 most deprived LSOAs in England across both the Townsend score and the IMD for all data cuts (i.e., Census or IMD years). This was derived by identifying the mean average rank for all LSOAs. As an example, the top ranked LSOA by the Townsend score (Tower Hamlets 015D) is ranked 81 in 1971, 27 in 1981, 5 in 1991, 2 in 2001, and 22 in 2011, with a mean average of 27.4 – the smallest mean for any LSOA and thus the most deprived across all Census time points. In the case of the TS, LSOAs in London dominate the top ten (seven out of ten) while for the IMD, the north west dominates (seven out of ten). As before, this reflects the larger weighting of household overcrowding in the TS relative to the IMD.

**Table 5:** The top 10 most deprived LSOAs for all years by (a) TS, (b) IMD

LSOA	Region	TS 71	TS 81	TS 91	TS 01	TS 11	Mean TS	IMD 04	IMD 07	IMD 10	IMD 15	IMD 19	Mean IMD
(a) Top 10 by maximum TS rank													
Tower Hamlets 015D	London	81	27	5	2	22	27.4	663.5	564	395	2408.5	4749	1756
Tower Hamlets 021A	London	232	8	2	26	64	66.4	1135	906	748.5	4078	6845	2742.5
Liverpool 039D	North West	255	42	45	13	13	73.6	95	52	51	210	511	183.8
Tower Hamlets 008A	London	96	69	93	27	87	74.4	1461.5	1949	3556.5	4738	6856	3712.2
Tower Hamlets 015E	London	112	115	1	33	123	76.8	863.5	588	511	4509	5125	2319.3
Liverpool 037D	North West	54	25	19	83	278	91.8	304	195	193.5	695	1512	579.9
Kensington and Chelsea 001E	London	265	108	42	17	42	94.8	3667	2939	2915	1243	1012	2355.2
Newcastle upon Tyne 029A	North East	26	125	9	64	257	96.2	287.5	435	998	1457	1631	961.7
Hammersmith and Fulham 002D	London	298	123	53	85	68	125.4	3568.5	2873	3296	3649	3771	3431.5
Lambeth 007B	London	186	68	146	150	114	132.8	3469.5	2387	3850	2785	6491	3796.5
(b) Top 10 by maximum IMD rank													
Tendring 018A	East	4818	9068	6189	4428	3757	5652	105	4	1	1	1	22.4
Manchester 009G	North West	53	106	164	1843.5	256	484.5	1.5	2.5	10.5	32	79	25.1
Wirral 011C	North West	169	310	1015	672	835	600.2	23	24	24	36	19	25.2
Rochdale 010C	North West	3974	3060	995	1042	214	1857	16	14	71	33	31	33
Liverpool 018F	North West	1630	2746	965	951	782	1414.8	6	8	8	29	114	33
Liverpool 024B	North West	1767	448	433	776	475	779.8	38	31	41	44	43	39.4
Knowsley 008F	North West	41	21	85	440	1299	377.2	10	16	53	69	56	40.8
North East Lincolnshire 002B	Yorkshire and The Humber	1367	1264	447	495	132	741	65.5	20	16	25	104	46.1
Blackpool 007C	North West	1194	814	718	1410	896	1006.4	129	5	4	42	52	46.4
Middlesbrough 003F	North East	1278	1061	2265	331	878	1162.6	60	29	29	64	51	46.6

**Fig. 5:**
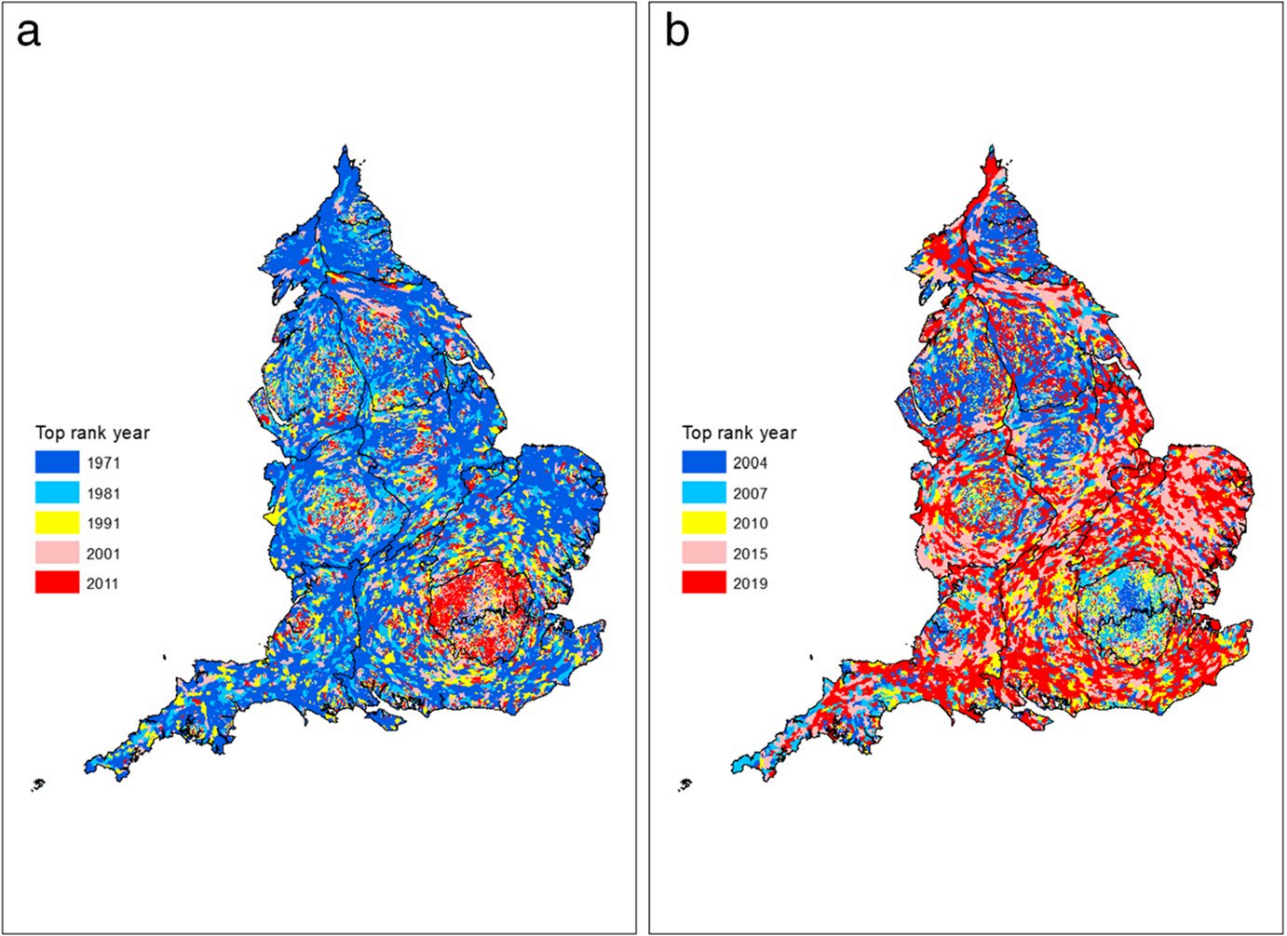
Year in which each LSOA had its highest deprivation ranking (the most deprived) by (**a**) Townsend score, (**b**) IMD

Another way of summarising deprivation patterns across time is to identify the year in which the Townsend score was largest and the year the IMD were largest. Figure 5a shows that in many (especially rural) areas the Townsend score was largest in 1971. In many areas of the south and east of England the Townsend score was largest in 1991. The most obvious concentration of LSOAs with the largest Townsend value in 2001 and 2011 is a ring encompassing the outer-London boroughs and these are associated with high and growing levels of overcrowding (Lloyd & Gleeson, 2022; see also the discussion above). For the IMD (Fig. 5b), parts of many urban areas (including central London), had their largest values in 2004 while in most rural areas, particularly in the southern half of England, the largest scores were found in 2015. There are some areas with their largest scores in proximate data cuts for both scores—for example, LSOAs in parts of outer London had their largest Townsend score in 2011 and their largest IMD score in 2007 or 2010.

Some 1,352 LSOAs are in the top TS deprivation deciles for all five Census time points. There are 1,831 LSOAs in the top IMD decile for all five timepoints. In total, 667 LSOAs are in the top decile for both the TS and IMD at all ten time points. Some 1,138 LSOAs are in the top decile for all but one of the measures and in the top two deciles for the remaining one. There are 2,565 LSOAs in one of the top two deciles for both the TS and IMD at all time points. This suggests a considerable amount of continuity across measures and time points.

## Claimant Count and Deprivation

**Fig. 6:**
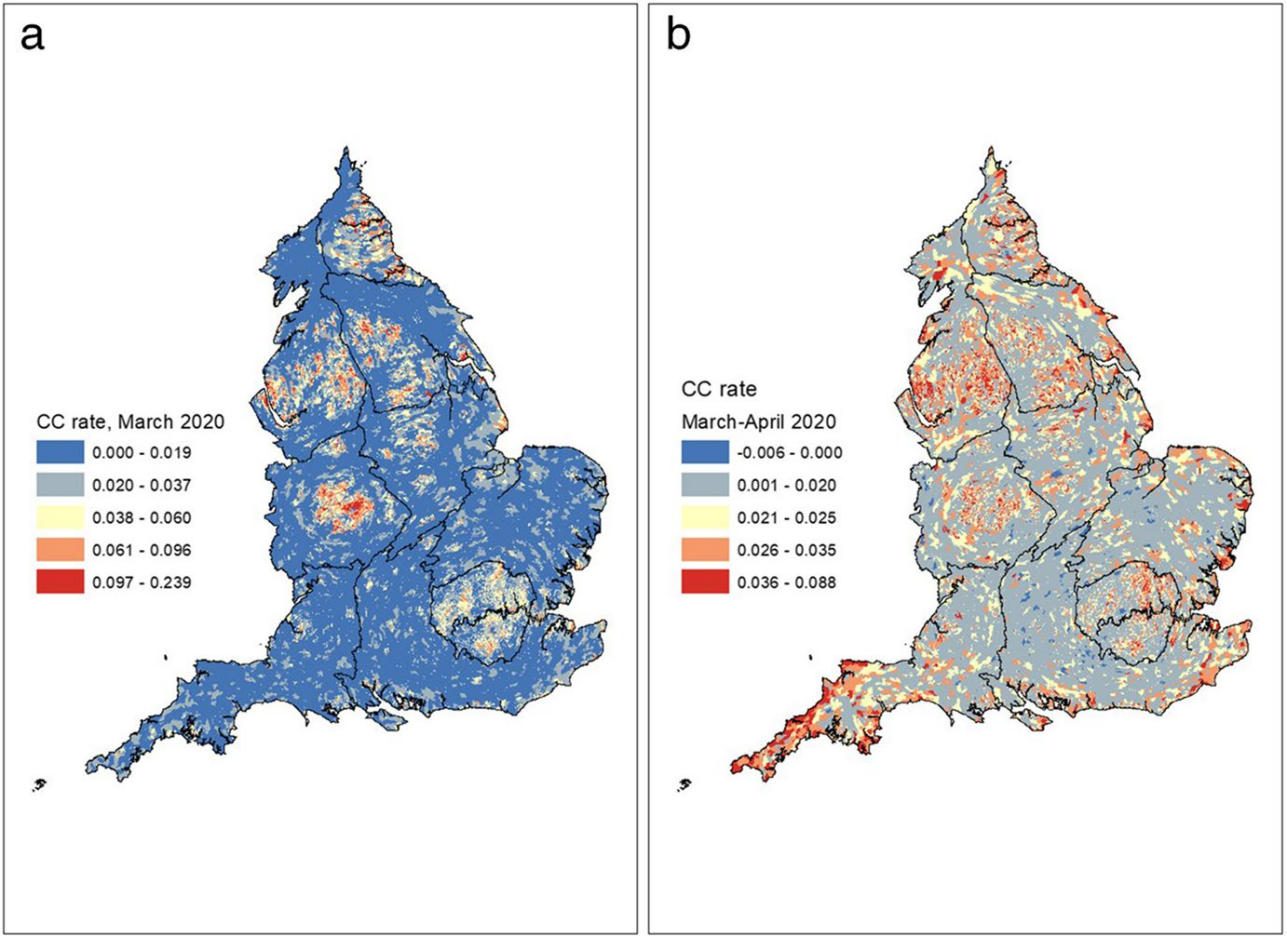
Claimant count rate in (**a**) March 2020, (**b**) difference between March 2020 and April 2020, where positives are increases

The final strand of the analysis considers how unemployment has changed following the first Covid-19 national lockdown. Connections between changes in unemployment and deprivation and unemployment prior to lockdown are assessed and this component of the paper contributes to a growing literature on the widening of inequalities in the face of the pandemic. Claimant count rates for March 2020 are shown in Fig. 6a while changes between March 2020 and April 2020 are mapped in Fig. 6b. Many of the largest increases in claimant count rates were in areas which had high rates before the pandemic, mirroring UK-wide research indicating that job losses are impacting most on low income groups and young people (Adams-Prassl et al., 2020). It is important to be aware that the UK government furlough scheme will have affected the numbers of people included in the claimant count and this is likely to have had an uneven impact geographically. Given that the highest levels of furlough take up were in particular employment sectors (most notably accommodation and food service activities, and arts, entertainment and recreation (ONS, 2020)), the composition of the workforce will have been a determinant of furlough and therefore have had an effect on unemployment.

Some 72% of LSOAs in the top IMD19 decile were also in the top 10% by claimant count rates in April 2020, while 94% of LSOAs in the top IMD19 decile were also in the top 20% by claimant count rates in April 2020. In other words, the majority of those areas which are considered highly deprived based on IMD19 also had very high claimant count rates (and thus high unemployment) immediately following the first national lockdown.

Assessing change in estimated unemployment levels is also important. Some 34% of LSOAs in the top IMD19 decile were in the top 10% of LSOAs by increase in claimant count rate between March and April 2020. This means that many of those neighbourhoods considered most deprived based on IMD19 had the highest rates of increase in claimant counts (and unemployment) between March and April 2020. It is worth nothing that the equivalent figures for May 2020 are smaller (66% in IMD top decile and May claimant count rate top decile; nearly 28% in top IMD19 decile and top decile by increase in claimant count rate between March and May 2020). This suggests that the impacts of job losses started to be felt in a wider array of communities as lockdown progressed. Nonetheless, the association between deprivation pre-lockdown and job losses following the start of lockdown is clear. This provides strong support for the contention that Covid-19 has exacerbated existing inequalities.

## Discussion and Conclusions

This paper details a new resource which links Census-based variables and IMD scores to a common geography (LSOAs). It is demonstrated that deprivation patterns (using alternative definitions of deprivation) are relatively consistent across time. The core of most urban areas are in the highest deprivation deciles for all measures and at all time points. The Townsend score is obviously different in concept and contents than the IMD, but the degree of similarity in the trends the two measures capture suggests that the Townsend score can usefully provide context for the IMD. A deprivation time series is valuable on the grounds that the population of a LSOA (for example) with a long history of deprivation but high IMD score in 2019 is likely to require different strategies than one with a shorter history of deprivation but an equally high IMD 2019 score. Thus, the core rationale behind the paper is that understanding deprivation *change*, and not just the level of deprivation captured for the most recent period, is important. Deprivation persistence is important in assessing the likely success of schemes to reduce inequalities (see JRF 2014 for a discussion of this theme).

McLennan et al. (2012) have produced an ‘Economic Deprivation Index’ (EDI) for 1999–2009. The EDI consists of two domains: an Income Deprivation Domain and an Employment Deprivation Domain and these are strongly related to the equivalent IMD domains. The EDI is backwards compatible and so can be used to explore changes across time. In addition, the individual domains can be used to explore absolute and not just relative change in deprivation. This index could be replicated for the post 2009 period and linked to Census-based measures and this would enable an even more detailed assessment of deprivation changes at the local level (albeit with a more restricted notion of deprivation than that provided by the IMD). Given that the Townsend score variables can, like the EDI, be used to measure absolute change, combining these with the EDI would offer the means to explore how far long term deprivation increases have, for example, a function of differential rates of improvement (all places improve, but some less than others and thus they fall behind), or increases in absolute deprivation. This is crucial in unpicking the nature of changes in deprivation locally. In addition, the approach of Abel et al. (2016) could be used to adjust the income and employment domains of the IMD allowing comparisons between the four nations of the UK. It is worth noting that developing a measure of change over time on a consistent basis using administrative data is challenging due to the constantly evolving nature of benefit policies in the UK. This is a particular problem with the introduction of Universal Credit as the new benefit differs substantially from the legacy benefits in multiple ways (see McLennan et al., 2019; Powell, 2020).

The Townsend index and the IMD each capture important elements of changing deprivation levels in England. There are, however, significant components of deprivation and poverty not captured by these indices. In particular, most households which can be categorised as poor are now home to one or more adults who are in work. A growing body of research has demonstrated that unemployment alone provides an incomplete guide to deprivation and income poverty given the transitions between unemployment benefits and sickness and disability benefits observed over the last 20 years and the rise of in-work poverty (see, for example, Hick & Lanau, 2018; Bourquin et al., 2019).

A nuanced assessment of different forms of poverty and wealth is provided by Dorling et al. (2007) who use a range of data sources (including the Census and the Family Expenditure Survey) to determine how many households were (1) core poor (the poorest), (2) breadline poor (households who lack material resources which exclude them from the norms of society), (3) asset wealthy (those who will expect to pay inheritance tax on the basis of their housing wealth), or (4) exclusive wealthy (those who can use their wealth to exclude themselves from the norms of society). The study was undertaken in recognition of the fact that poor households are not a homogenous group and that income may not properly reflect social divides in a context where the wealthiest may not need to draw a conventional income through paid employment, while many people in the poorest group may not be able to work and have little income. In the element of their study focusing on the post 2000 period, Dorling et al. (2007) considered spatial trends in JSA and IB claimant rates. They also estimated average income for temporally consistent areas (termed ‘tracts’, with a mean population of around 45,000 people) using data provided by Barclays Bank. In contrast to the JSA claimant rate changes, which suggest decreasing spatial inequalities, there was evidence for growing income spatial inequality – areas with the highest average incomes in 2003 saw the largest increases to 2005. The authors argue that “The relationship between JSA claimant rates and actual worklessness is not necessarily straightforward, but, this aside, the contradictory findings between the JSA and income analyses may indicate that lack of paid employment is becoming a less useful indicator of poverty” (Dorling et al., 2007, p. 86).

Dorling et al. (2007) estimate mean income; the IMD income domain says nothing about actual incomes in an area, other than whether they are above/below the equivalised income poverty threshold. As such, we cannot determine whether income inequality is increasing or decreasing or changing given the IMD income domain. There are, therefore, several important limitations of the present analysis and important drivers of local changes that this analysis will miss. There is scope to expand the analyses presented here to more fully unpick the changing components of deprivation, potentially making use of an increasing array of accessible administrative data sources.

The paper builds on a major body of work by Paul Norman and colleagues which assesses change in deprivation between 1971 and 2011 (e.g., Norman, 2016) and considers how composite deprivation indicators could be constructed using administrative data (Ajebon & Norman, 2016). This key contribution here is in (i) linking to the IMD for four time points, (ii) identifying major components of change in deprivation, and (iii) assessing how the persistence of deprivation may be important. There is scope to expand all of these themes to, for example, include more variables (from the Census and administrative sources) and indices (such as the EDI up to 2009, and equivalents for more recent years), to model transitions in deprivation states with the addition of data on migration into and out of deprived areas, and to consider alternative characteristics which may relate to deprivation change (e.g., housing markets, changes in the numbers of single person households, employment diversity, etc.). As an example, long-term unemployment is spatially concentrated, especially following economic restructuring, but there is much debate about how far the persistence of unemployment should be tackled (see Lindsay, 2010). A stronger evidence base on local-level trends in unemployment with an array of additional variables has the potential to help unpick the determinants of long-term unemployment, as well as deprivation more generally. Rae et al. (2016), building on Robson et al. (2008), assess disconnection from local labour markets and housing markets (using migration and commuting data drawn from the Census) for areas in the most deprived 20% in each nation of the UK. Linking area histories to disconnection could offer additional insights into the impediments facing deprived communities.

 The findings from this paper are being used to create a series of deprivation change profiles for every local authority in England and the information contained in these, which includes detailed assessments of changes in individual domains of deprivation, will hopefully prove invaluable for LA analysts seeking to understand area change and as an evidence base for shaping policies aimed at tackling persistent deprivation. The results of similar analyses in Belfast, Northern Ireland (see Lloyd, 2022), are being used to guide targeting of employment training schemes. Including deprivation change alongside deprivation measures using recent data is vital if the distinct challenges of local areas are to be understood and addressed.

## Footnotes

1 http://ocsi.uk/2011/03/24/why-the-imd-is-still-important-in-the-open-data-age/

2 https://www.ons.gov.uk/employmentandlabourmarket/peoplenotinwork/outofworkbenefits/methodologies/claimantcount

3 https://www.gov.uk/jobseekers-allowance

4 https://popchange-data.liverpool.ac.uk/

5 Enumeration districts are geographical areas (with a mean population of some 450 people) which were used for data collection for the Censuses of 1971, 1981, 1991, and 2001. They were also used to provide data outputs from the Censuses in 1971, 1981, and 1991. https://www.ons.gov.uk/methodology/geography/ukgeographies/censusgeography#enumeration-district-ed

6 http://webarchive.nationalarchives.gov.uk/20160110195753/http://www.ons.gov.uk/ons/external-links/other/2001-lower-layer-super-output-areas--lsoa--to-2011-lsoas-and-lads.html

7 For 1971: working/(working + seeking work); for 1981–2011 inclusive: (employees + self-employed)/ (employees + self-employed + unemployed); full time and part time are listed separately for 1991 onwards and both are included in the counts used in the analyses.

8 The ‘Right to buy’ scheme enables some social housing tenants to purchase the property they live in at a reduced cost; see https://www.gov.uk/housing-local-and-community/homebuying

9 SDA and IB were benefits for people who have limited capability for work. These schemes have been replaced with Employment and Support Allowance (ESA); see https://www.gov.uk/incapacity-benefit

10 It is worth noting that the OAC also has a 'time stamp' and so will be more closely aligned with the 2011 outputs than the earlier outputs.
